# Increase in Tau Pathology in P290S *Mapt* Knock-In Mice Crossed with *App*^NL-G-F^ Mice

**DOI:** 10.1523/ENEURO.0247-22.2022

**Published:** 2022-12-19

**Authors:** Melissa Huang, Jennifer Macdonald, Isabelle Lavenir, Renren Chen, Molly Craxton, Elizabeth Slavik-Smith, Stephen W. Davies, Michel Goedert

**Affiliations:** 1 MRC Laboratory of Molecular Biology, Cambridge CB2 0QH, United Kingdom; 2 Department of Cell and Developmental Biology, University College London, London WC1E 6BT, United Kingdom

**Keywords:** Alzheimer’s disease, amyloid-β, knock-in, mouse model, tau

## Abstract

Alzheimer’s Disease (AD) is characterized by the pathologic assembly of amyloid β (Aβ) peptide, which deposits into extracellular plaques, and tau, which accumulates in intraneuronal inclusions. To investigate the link between Aβ and tau pathologies, experimental models featuring both pathologies are needed. We developed a mouse model featuring both tau and Aβ pathologies by knocking the P290S mutation into murine *Mapt* and crossing these *Mapt*^P290S^ knock-in (KI) mice with the *App*^NL-G-F^ KI line. *Mapt*^P290S^ KI mice developed a small number of tau inclusions, which increased with age. The amount of tau pathology was significantly larger in *App*^NL-G-F^*xMapt*^P290S^ KI mice from 18 months of age onward. Tau pathology was higher in limbic areas, including hippocampus, amygdala, and piriform/entorhinal cortex. We also observed AT100-positive and Gallyas-Braak-silver-positive dystrophic neurites containing assembled filamentous tau, as visualized by *in situ* electron microscopy. Using a cell-based tau seeding assay, we showed that Sarkosyl-insoluble brain extracts from both 18-month-old *Mapt*^P290S^ KI and *App*^NL-G-F^*xMapt*^P290S^ KI mice were seed competent, with brain extracts from double-KI mice seeding significantly more than those from the *Mapt*^P290S^ KI mice. Finally, we showed that *App*^NL-G-F^*xMapt*^P290S^ KI mice had neurodegeneration in the piriform cortex from 18 months of age. We suggest that *App*^NL-G-F^*xMapt*^P290S^ KI mice provide a good model for studying the interactions of aggregation-prone tau, Aβ, neuritic plaques, neurodegeneration, and aging.

## Significance Statement

Alzheimer’s disease (AD) is characterized by the presence of extracellular amyloid β (Aβ) plaques and intracellular neurofibrillary tangles made of filamentous tau. The interactions between Aβ and tau pathology remain unclear. We developed a knock-in mouse model of tau pathology, expressing mutant (P290S) murine tau under its natural promoter, which exhibited a small number of tau inclusions. When these mice were crossed with a knock-in model of Aβ pathology, we observed a significant, age-dependent increase in tau pathology, along with the appearance of other key features of AD, including dystrophic neurites surrounding Aβ plaques, seed-competent tau, and neurodegeneration. This suggests that this model will be useful for future studies investigating the interactions between tau and Aβ pathologies.

## Introduction

Alzheimer’s disease (AD) is defined by the simultaneous presence of two different filamentous amyloid inclusions: abundant extracellular deposits of amyloid β (Aβ) and abundant intraneuronal inclusions of tau. Genetic evidence has indicated that Aβ is key to the pathogenesis of Alzheimer’s disease (Hardy and Higgins, 1992; Hardy and Selkoe, 2002). Multiplications of the gene encoding the Aβ precursor protein (*APP*), as well as mutations in *APP* and the presenilin genes (*PSEN1* and *PSEN2)*, cause familial Alzheimer’s disease with abundant Aβ deposits and tau inclusions. It follows that both proteopathies are probably linked.

To investigate this link, one needs experimental systems that develop both pathologies. Although multiple transgenic mouse models with abundant Aβ deposits have been produced, filamentous tau inclusions did not form (Radde et al., 2006; Saito et al., 2014). Conversely, overexpression of human mutant tau can result in the development of filamentous tau inclusions and neurodegeneration, but in the absence of Aβ deposits (Allen et al., 2002). Crossing mice that overexpress human mutant APP with mice overexpressing human mutant tau has been shown to result in more assembled tau, suggesting that Aβ exacerbated tau assembly (Lewis et al., 2001; Chen et al., 2016). Moreover, in 3xTg-AD mice, which express three mutations in human APP, tau, and PSEN1, a reduction in Aβ deposits by immunotherapy resulted in decreased tau inclusions (Oddo et al., 2004).

While these models show a potentially significant interaction between tau and Aβ pathologies, they can suffer from artifactual phenotypes as a result of uncontrolled expression and/or random genomic insertion of the transgenes. Thus, the overexpression of APP can result in elevated levels of fragments that are not overexpressed in diseased human brains. Moreover, insertion of the transgenes may lead to the interruption of genes that are vital for nerve cell function (Saito et al., 2016; Gamache et al., 2019).

Knock-in (KI) models can provide a way around these drawbacks, since expression is driven by the natural promoters. In recent years, mouse lines were produced that deposit large amounts of human Aβ, without requiring overexpression of APP (Saito et al., 2014). Knock-in models, however, have so far not led to the formation of abundant filamentous tau deposits. Only models of the overexpression of multiple human wild-type tau isoforms or single-mutant human tau isoforms have given rise to abundant tau filaments and neurodegeneration (Ishihara et al., 1999; Allen et al., 2002; Sahara et al., 2002). A murine knock-in line that expresses all six humanized brain tau isoforms did not develop filamentous tau inclusions on crossing with the *App*^NL-G-F^ KI line, which expresses humanized Aβ, together with the Swedish (KM670/671NL), Arctic (E693G), and Beyreuther/Iberian (I716V) mutations (Saito et al., 2019). Similarly, a mouse line with a P301L tau knockin, did not exhibit filamentous tau inclusions (Gilley et al., 2012).

Here we describe the production and characterization of a mouse model of tauopathy, where mutation P290S was knocked into exon 10 of murine *Mapt*, the tau gene. Murine P290S tau is equivalent to human P301S tau that causes dominantly inherited frontotemporal dementia and results in the formation of abundant filamentous tau inclusions and neurodegeneration when overexpressed in transgenic mice (Bugiani et al., 1999; Allen et al., 2002; Yoshiyama et al., 2007). *Mapt*^P290S^ knock-in mice developed small numbers of tau inclusions that increased with age. To develop a model for the relationships between Aβ and tau pathologies, we crossbred *Mapt*^P290S^ knock-in mice with line *App*^NL-G-F^. These mice developed both Aβ and filamentous tau pathologies, and exhibited a significant and age-related increase in the number of tau inclusions when compared with *Mapt*^P290S^ knock-in mice.

## Materials and Methods

### Mouse lines

**Figure 1.**
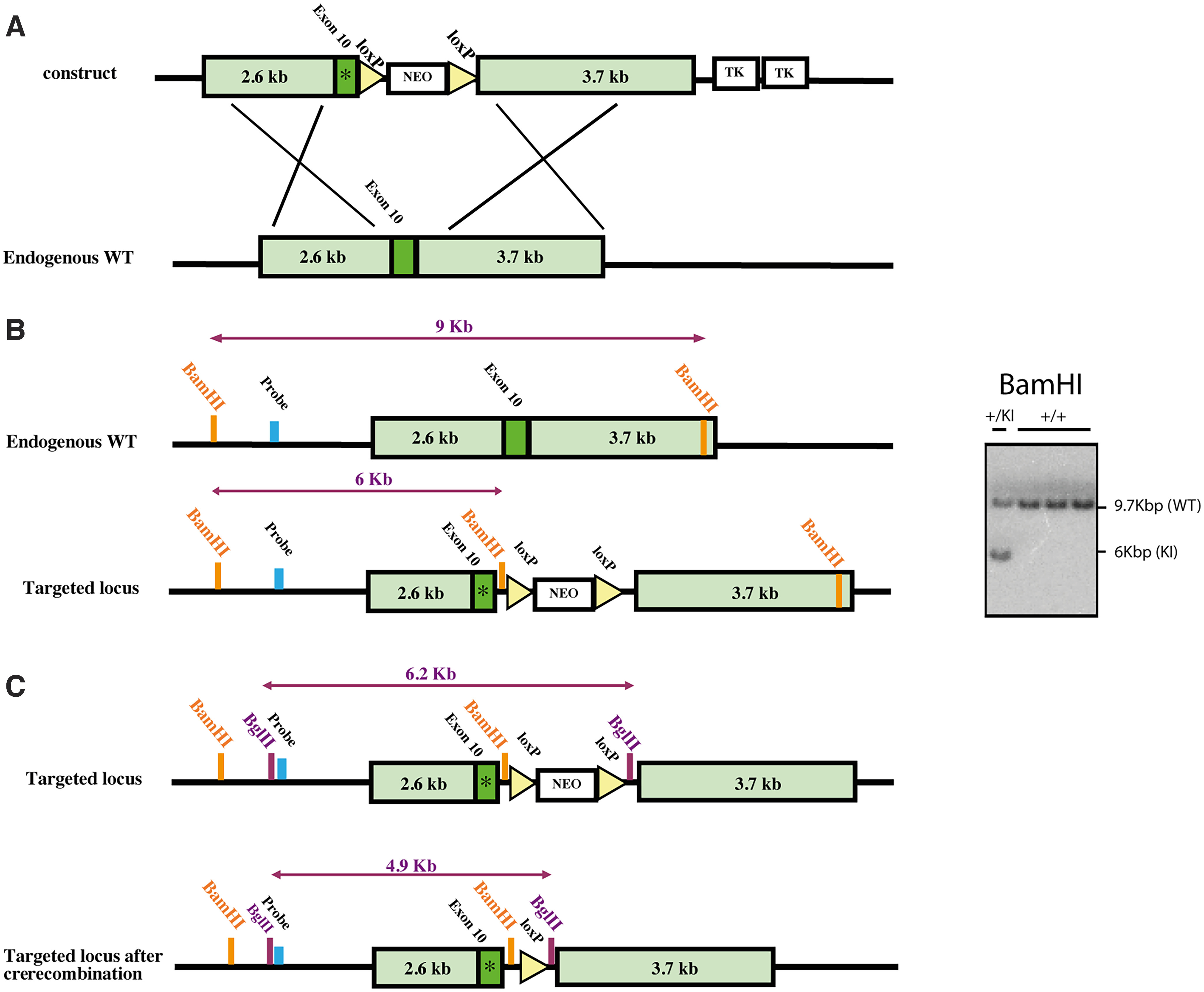
Generation of the *Mapt*^P290S^ KI mice. ***A***, Outline of the targeting strategy for introducing the murine equivalent of the P301S mutation (P290S) into exon 10 of the murine *Mapt* gene. ***B***, Screening strategy for selection of positive ES clones. Southern blot analysis of BamHI-digested genomic DNA. ***C***, Removal of the Neo positive selection marker by crossing the line with Stella-Cre transgenic line. *Indicates the P290S mutation.

Animal experiments were conducted in accordance with the UK Animals (Scientific Procedures) Act of 1986, with local ethical approval (MRC Laboratory of Molecular Biology Animal Welfare and Ethical Review Body). To generate the *Mapt*^P290S^ knock-in line, a targeting construct was designed to insert S290 into exon 10 of *Mapt*, the murine tau gene, by homologous recombination (Fig. 1). The targeting construct was transfected into calcium channel blocker embryonic stem cells (129 S/Sv) and positive clones were identified by Southern blotting. They were injected into C57BL/6 blastocysts, and the resulting chimeras were crossed with C57BL/6 mice, to establish germline transmission. The progeny was genotyped by PCR (oligos for wild-type allele, *CAGCAAAGTAGGGAGAGCAAC* and *CAGAGATGAGGGAAGAGGTGTC*; oligos for knock-in allele, *CAGCAAAGTAGGGAGAGCAAC* and *TTCGCCAATGACAAGACGC*). The Neo-loxP cassette was removed by crossing with Stella-Cre transgenic mice that express Cre recombinase (Liu et al., 2011). The presence of murine tau with the P290S mutation (equivalent to human P301S tau) was confirmed by DNA sequencing. Following backcrossing, analysis using single nucleotide polymorphisms established that the line was on a 99.9% C57BL/6 background (The Jackson Laboratory; MAXBAX 384 SNP panel, Charles River). *App*^NL-G-F^ knock-in mice express humanized Aβ and carry the Swedish double mutation (KM670/671NL) in APP, the Arctic mutation in Aβ (E22G) and the Beyreuther/Iberian mutation (I716F) in APP (Saito et al., 2014). The strain was generated using C57BL/6N ES cells. The line was backcrossed for eight generations to establish the C57BL/6J-based knock-in strain, which is now 99% C57BL/6J background (Takashi Saito, personal communication). To generate the *App*^NL-G-F^*xMapt*^P290S^ knock-in line, mice from the *Mapt*^P290S^ knock-in line were crossbred with mice from the *APP*^NL-G-F^ knock-in line. All lines were made homozygous by genotyping of the progeny by PCR.

### Tissue preparation

Tissues were collected from mice 3, 6, 12, 18, and 22–24 months of age (*n* = 4–8/time point). The sexes of the species used in each experiment have been designated as male (M) or female (F) in the Materials and Methods section and figure legends below. They were perfused intracardially with 4% paraformaldehyde in 0.1 m PBS, pH 7.4. Brains were dissected and postfixed overnight at 4°C, followed by cryoprotection in 20% sucrose in PBS for at least 24 h. Coronal sections (50 μm) were cut on a freezing microtome (model SM2400, Leica Microsystems) and kept at 4°C in PBS with 0.1% sodium azide.

### Immunostaining

All steps were performed on a rocker at room temperature, with the exception of primary antibody incubation, which was performed at 4°C. Wash steps were carried out in PBS containing 0.1% Triton X (PBST), three times for 10 min each. For immunohistochemistry, endogenous peroxidase activity was quenched by incubation with 0.3% hydrogen peroxide in water for 30 min, followed by washing. Sections were incubated in blocking buffer consisting of either 10% normal goat serum or horse serum in PBST for 1 h, followed by an overnight incubation with primary antibodies diluted in blocking buffer. The next day, sections were washed and incubated in biotinylated horse anti-mouse IgG or goat anti-rabbit IgG secondary antibody (1:200; Vector Laboratories) for 1 h, followed by washing. Sections were incubated with the VECTASTAIN ELITE ABC Kit (Vector Laboratories) for 1 h, followed by washing, and developed with a diaminobenzidine peroxidase substrate kit (Vector Laboratories). Immunostained sections were then washed in water, mounted onto Superfrost plus slides (Thermo Fisher Scientific), air dried, and coverslipped with Pertex mounting medium (CellPath).

For immunofluorescence, before incubation with a primary antibody made in mouse (AT100), sections were incubated in goat F(ab) anti-mouse IgG (1:10; catalog #ab6668, Abcam) overnight at 4°C to block endogenous mouse IgG. Following primary antibody incubation overnight at 4°C, sections were washed and incubated in Alexa Fluor 647-conjugated secondary antibody (1:500; catalog #A-21245, Thermo Fisher Scientific) or Alexa Fluor 488-conjugated streptavidin (1:500; catalog #S11223, Thermo Fisher Scientific) diluted in PBST for 1 h at room temperature. After immunolabeling, sections were washed and counterstained with DAPI (1:1000 in distilled H_2_O) for 5 min. Lipofuscin autofluorescence was quenched using TrueBlack lipofuscin autofluorescence quencher (catalog #23007, Biotium) diluted 1:20 in 70% ethanol, and sections were washed 5–6× in PBST before being mounted onto slides. Slides were coverslipped with VECTASHIELD Anti-Fade Mounting Medium (catalog #H-1000–10, Vector Laboratories), and the edges were sealed with nail varnish for storage at 4°C. Images were acquired on an inverted confocal microscope (model 780, Zeiss).

### Image quantitation

Cells immunoreactive with anti-tau antibodies AT8 and AT100 in a 1:24 series of sections (eight sections per brain) were counted manually from frontal pole to locus coeruleus in ImageJ, facilitated by the Cell Counter plug-in. Cells with a defined cell body and one or more connecting processes were counted. Regional quantitation of AT8-positive cells was performed following the Franklin and Paxinos (2013) brain atlas. Cells were counted in five brain regions [paraventricular thalamic nucleus (PV), periaqueductal gray (PAG), hippocampus, amygdala, and piriform cortex] in 1:3 serial sections (eight sections per brain). Sections were imaged using a microscope (model Ti2, Nikon) and analyzed in ImageJ. Regional quantitation depicts the average number of immunoreactive cells per pixel area measured. For quantitation of AT8 staining in brains of *Mapt*^P290S^ KI mice at the following times: 3 months, *n* = 6 (3 M, 3 F); 6 months, *n* = 6 (3 M, 3F); 12 months, *n* = 8 (4 M, 4 F); 18 months, *n* = 8 (4 M, 4 F); 22–24 months, *n* = 6 (3 M, 3 F). In *App*^NL-G-F^*xMapt*^P290S^ KI mice at the following times: 3 months, *n* = 6 (3 M, 3 F); 6 months, *n* = 8 (4 M, 4 F); 12 months, *n* = 8 (4 M, 4 F); 18 months, *n* = 8 (4 M, 4 F); 22–24 months, *n* = 4 (2 M, 2 F). Brain sections from 4 (2M, 2F) 18-month-old *App*^NL-G-F^*xMapt*^P290S^ KI mice were not available for AT100 staining. For regional quantitation, brain sections were available at: 3, 6, 12, and 18 months, *n* = 6 (3 M, 3 F); and 22–24 months, *n* = 4 (2 M, 2 F).

### Gallyas-Braak silver staining

Eight sections (1:24 series) per brain were mounted onto slides and left to air dry. Reagents were prepared according to the study of Braak and Del Tredici (2015). Slides with mounted sections were pretreated with 3% periodic acid for 10 min, followed by a 3 min wash in distilled water. They were then transferred to alkaline solution of silver iodide complexes for 2 min. Slides were immersed in developer for 30–50 min. Following this, they were immersed in the following: 0.5% acetic acid (3 min), distilled water (3 min), gold chloride (1 min), distilled water (3 min), 0.5% sodium thiosulfate (5 min), and distilled water (3 min). Silver staining was performed on brains sections from mice [at 3 and 6 months, *n* = 3 (2 M, 1 F); 12 and 18 months, *n* = 8 (4 M, 4 F); and 22–24 months, *n* = 4 (2 M, 2 F)].

### Extraction of Sarkosyl-insoluble tau from mouse brain homogenates

Fresh frozen whole mouse brains were weighed and homogenized in 10% (w/v) extraction buffer (20 mm Tris-HCl, pH 7.4, 800 mm NaCl, 5 mm EDTA, 15% sucrose, 1% Sarkosyl, and one tablet/10 ml complete protease/phosphatase inhibitor cocktail; Pierce), and total protein was normalized using the Pierce BCA protein assay kit (Thermo Fisher Scientific). Sarkosyl-insoluble tau was extracted by differential centrifugation as described previously (Goedert et al., 1992). Briefly, tissue homogenates were incubated for 1 h in 1% Sarkosyl and centrifuged at 18,000 × *g* for 20 min at room temperature. Supernatants were filtered through a 45 μm cell strainer, then spun for 1 h at room temperature at 45,000 rpm using a TLA-55 rotor in an Optima Max XP ultracentrifuge. Pellets were resuspended in 150 mm NaCl, and 50 mm Tris HCl, at pH 7.4, and spun again at 45,000 rpm. The final pellets were resuspended in 30 μl of 150 mm NaCl, 50 mm Tris HCl.

### Seeding assay

HEK293 cells stably expressing human 0N4R P301S tau-venus (McEwan et al., 2017) were maintained in DMEM plus GlutaMAX (catalog #31966–021, Thermo Fisher Scientific) with 10% fetal calf serum (catalog #10270–106, Thermo Fisher Scientific) and 1% penicillin/streptomycin. Black 96-well plates (catalog #3603, Costar) were pretreated with poly-d-lysine, and HEK293 P301S tau-venus cells were plated at a density of 20,000 cells/well and allowed to adhere overnight. Cells were then washed with PBS, and 50 μl of prepared tau filaments extracts, or seeds, diluted 1:50 in OptiMEM (catalog #3198502, Thermo Fisher Scientific) with 1:50 lipofectamine 2000 (catalog #11668027, Thermo Fisher Scientific), was added to each well. Cells were incubated with seeds for 1 h before adding 100 μl of complete-DMEM to block further entry of lipofectamine–seed complexes. After 48 h, cells were rinsed with PBS and fixed with 4% paraformaldehyde for 10 min at room temperature. For immunohistochemistry, cells were incubated in primary antibody overnight at 4°C, followed by washing and incubation in Alexa Fluor 647-conjugated goat anti-mouse IgG secondary antibody (1:500; Thermo Fisher Scientific) for 1 h at room temperature. Cells were then incubated with 1 μg/ml Hoechst 33342 diluted in 1× PBS for 10 min. Images were taken using an inverted fluorescence microscope (model HCA, Nikon) under a 10× lens. Image analysis was performed using NIS-Elements AR. Quantitation represents the total area of the venus puncta signal per nucleus. Immunofluorescence images were taken on an inverted confocal microscope (model 780, Zeiss) under a 20× lens.

### Extraction of Sarkosyl-insoluble tau from seeded cells

HEK293 P301S tau-venus cells were plated at 500,000 cells/well in six-well plates and allowed to adhere overnight. Sarkosyl-insoluble tau filament extracts were prepared at 1:50 in 500 μl of OptiMEM containing 1:50 lipofectamine 2000 (catalog #11668027, Thermo Fisher Scientific) and added to 500 μl of cells in DMEM. After 1 h, 1 ml of DMEM was added to stop seeding. After 48 h, cells were washed gently with PBS before being resuspended by forceful pipetting in 2 ml of PBS. Resuspended cells were centrifuged at 400 × *g* for 5 min, and the resultant pellets were resuspended in 500 μl of extraction buffer (10 mm Tris, pH 7.4, 0.8 m NaCl, 1 mm EGTA, at pH 7.2, and 5 mm EDTA, at pH 7.4, and 10% sucrose) and lysed on ice for 30 min. Cell homogenates were centrifuged at 18,000 relative centrifugal force for 20 min at 4°C. Protein concentrations of the resultant supernatants were determined by the Pierce BCA Protein Assay Kit (catalog #23225, Thermo Fisher Scientific) and normalized to the lowest concentration. Supernatants were then spun at 45,000 rpm for 1 h at 4°C. The pellets were resuspended in 200 μl of 50 mm Tris HCl, at pH 7.4, and spun again as before. The final pellets were resuspended in 15 μl of 50 mm Tris, pH 7.4, and stored at 4°C until immunoblot analysis.

### Electron microscopy

Extracted tau filaments were diluted 10-fold to 20-fold and deposited on glow-discharged 400-mesh formvar/carbon film-coated copper grids (model CF400-Cu, EM Sciences) for 40 s. Grids were blocked for 10 min in 0.1% cold water fish skin gelatin in PBS before a 1 h incubation with primary antibodies diluted in blocking buffer. Grids were washed with blocking buffer and incubated with gold-conjugated anti-mouse IgG (catalog #G7652, Sigma-Aldrich) or anti-rabbit (catalog #G7402, Sigma-Aldrich) secondary antibody for 1 h. Following washing, the grids were stained with 4 μl of 2% uranyl acetate for 90 s. Images were acquired at 11,000× and 15,000×, with a defocus value of −1.4 mm using a transmission electron microscope (Tecnai G2 Spirit) at 120 kV. For *in situ* electron microscopy (EM), perfusion-fixed brains (4% paraformaldehyde) were postfixed in 4% paraformaldehyde/0.1% glutaraldehyde for at least 24 h. Sections (50–200 μm) were cut on an Oxford vibratome and collected in PBS. Gallyas-Braak silver staining was performed on vibratome-cut sections. Following osmication (30 min in 1% OsO_4_ in 100 mm phosphate buffer), the sections were stained for 15 min in 0.1% uranyl acetate in sodium acetate buffer at 4°C, dehydrated, cleared in propylene oxide, and embedded in Araldite resin between two sheets of Melanex (Imperial Chemical Industries). Semithin (1 μm) sections were cut with glass knives and stained with toluidine blue adjacent to thin sections (70 nm) that were cut with a diamond knife on a Reichert Ultracut ultramicrotome. Sections were collected on copper mesh grids, counterstained with lead citrate and viewed in an electron microscope (model 1400, Jeol). Photomicrographs were taken with a Gatan Rio Camera.

### Immunoblotting

Total, soluble, and Sarkosyl-insoluble protein samples of mouse brains and HEK293 P301S tau-venus-seeded cell homogenates were run on a Novex WedgeWell 4–20% Tris-Glycine gels (catalog #XP04205BOX, Thermo Fisher Scientific) and transferred onto PVDF membranes (catalog #1704156, BIO-RAD). The blots were then blocked in 5% bovine serum albumin in PBS plus 0.2% Tween and incubated overnight at 4°C with primary antibodies diluted in blocking buffer, followed by either goat anti-mouse (1:5000; catalog #1706516, BIO-RAD) or goat anti-rabbit (1:4000; catalog #1706515, BIO-RAD) HRP-conjugated secondary antibodies, Dylight 680 goat-conjugated anti-mouse (1:10,000; catalog #5470, Cell Signaling Technologies), or Dylight 800 4× polyethylene glycol-conjugated goat anti-rabbit (1:10,000; catalog #5151, Cell Signaling Technologies) secondary antibodies. Signal was visualized using enhanced chemiluminescence (GE Healthcare) with x-ray film or using near-infrared fluorescence detection (ChemiDoc MP, BIO-RAD), and band intensities were quantitated using ImageJ.

For immunoblot analysis, the *n* values were as follows at: 3 and 12 months, *n* = 3 (2 M, 1 F); and 6 and 18 months, *n* = 3 (1 M, 2 F).

### Stereology

Four sections (1:24 series) per brain were used for cell counting with SteroInvestigator 11 (MBF Bioscience). Section thickness was determined using Investigator software. For each section, a 200 × 400 μm region of piriform cortex was traced under a 5× objective, starting from the ventral side. NeuN-positive cells and their nuclei within the dissector volume were counted using the 100× objective. The investigator was blind with respect to the nature of the groups. For wild-type mice at all ages, *n* = 3 M; for *Mapt*^P290S^ KI mice at 12 and 18 months, *n* = 4 (2 M, 2 F), and at 22–24 months, *n* = 6 (3 M, 3 F); and for *App*^NL-G-F^*xMapt*^P290S^ KI at 12 and 18 months, *n* = 4 (2 M, 2 F), and at 22–24 months, *n* = 5 (2 M, 3 F).

### Statistics

Statistical analysis was performed using GraphPad Prism software. All the data are shown as the mean ± SEM. Data were analyzed by one-way or two-way ANOVA, followed by Tukey’s multiple-comparisons test.

### Antibodies

To detect tau by immunostaining, we used biotin-conjugated anti-pS202/pT205 tau (AT8; 1:500; mouse monoclonal, catalog #MN1020B, Thermo Fisher Scientific) and anti-pT212/pS214/pT217 tau (AT100; 1:500; mouse monoclonal, MN1060, Thermo Fisher Scientific). The following additional antibodies were used for immunostaining: anti-Aβ (D12B2; 1:1000; rabbit monoclonal; catalog #9888, Cell Signaling Technology) and anti-NeuN (1:500; mouse monoclonal; catalog #MAB377, Millipore). For immunoblotting, we used the following antibodies: anti-mouse tau (T49; 1:50,000; mouse monoclonal; catalog #MABN827, Millipore), anti-APP (22C11; 1:2500; mouse monoclonal; catalog #MAB348, Millipore), anti-GAPDH (1:10,000; mouse monoclonal; catalog #MAB374, Millipore), AT8 (1:500), AT100 (1:500), anti-pS422 tau (1:1000; rabbit monoclonal; catalog #ab79415, abcam), anti-tau (BR133; 1:4000; rabbit polyclonal, in-house), and anti-tau (BR134; 1:4000; rabbit polyclonal, in-house). For immunoelectron microscopy, we used the following antibodies: anti-mouse tau (MT1; 1:50; rabbit polyclonal, in-house), BR134 (1:50), AT8 (1:50), and AT100 (1:50).

## Results

### Tau filaments in Sarkosyl-insoluble fraction from *App*^NL-G-F^ KI*xMapt*^P290S^ KI line

**Figure 2.**
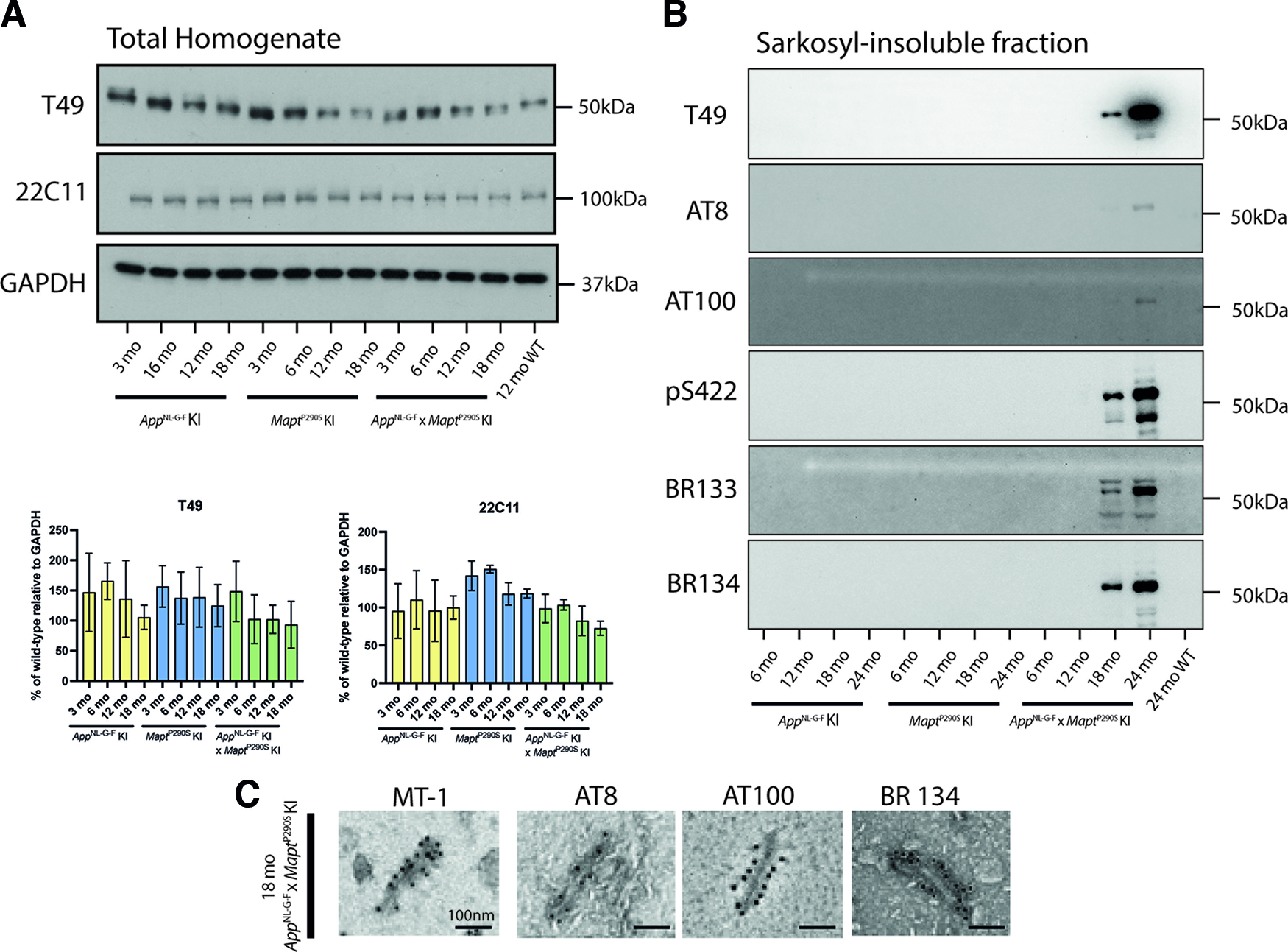
Immunoblot analysis and immuno-EM of Sarkosyl-insoluble brain extracts. ***A***, Quantitation and representative immunoblots of T49 and 22C11 expression in total homogenates from brains of 3-, 6-, 12-, and 18-month-old *App*^NL-G-F^, *Mapt*^P290S^, and *App*^NL-G^*^-^*^F^*xMapt*^P290S^ KI mice. At 3 and 12 months, *n* = 3 (2 M, 1 F). At 6 and 18 months, *n* = 3 (1 M, 2 F). ***B***, Representative immunoblots of Sarkosyl-insoluble tau from the brains of 6-, 12-, 18-, and 24-month-old *App*^NL-G-F^, *Mapt*^P290S^, and *App*^NL-G-F^*xMapt*^P290S^ KI mice using antibodies T49, AT8, AT100, pS422, BR133, and BR134. At 3 and 12 months, *n* = 3 (2 M, 1 F). At 6 and 18 months, *n* = 3 (1 M, 2F). ***C***, Immunoelectron microscopy of Sarkosyl-insoluble filaments of an 18-month-old *App*^NL-G-F^*xMapt*^P290S^ KI mouse brain.

We generated a knock-in mouse model of tau assembly (*Mapt*^P290S^ KI) by incorporating a mutation equivalent to P301S, which causes an inherited form of frontotemporal dementia in humans (Bugiani et al., 1999), into exon 10 of *Mapt* (Fig. 1; see Materials and Methods section). *App*^NL-G-F^ KI mice were used as a model of Aβ deposition. *Mapt*^P290S^ KI mice were crossed with *App*^NL-G-F^ KI mice, to generate a double-KI model (*Mapt*^P290S^ KI*xApp*^NL-G-F^ KI), with tau and Aβ pathologies. Immunoblot analysis (Fig. 2*A*) of brain homogenates from 3-, 6-, 12-, and 18-month-old mice from single- and double-KI lines showed no significant differences in expression levels of tau and APP between wild-type and KI mice. Sarkosyl-insoluble fractions from the brains of mice from the *App*^NL-G-F^ KI, *Mapt*^P290S^ KI, and *App*^NL-G-F^ KI*xMapt*^P290S^ KI lines were analyzed by immunoblotting. Only in the *App*^NL-G-F^ KI*xMapt*^P290S^ KI line did we detect Sarkosyl-insoluble tau. From 18 months of age, anti-tau antibodies T49, AT8, AT100, pS422, BR133, and BR134 detected a Sarkosyl-insoluble band of 55 kDa (Fig. 2*B*). Immunogold negative-stain electron microscopy showed abundant tau filaments in the Sarkosyl-insoluble fraction from the brains of mice from the *App*^NL-G-F^ KI*xMapt*^P290S^ KI line. Filaments were decorated by MT-1, AT8, AT100, and BR134 (Fig. 2*C*).

### Increase in whole-brain tau pathology in *App*^NL-G-F^ KI*xMapt*^P290S^ KI line

**Figure 3.**
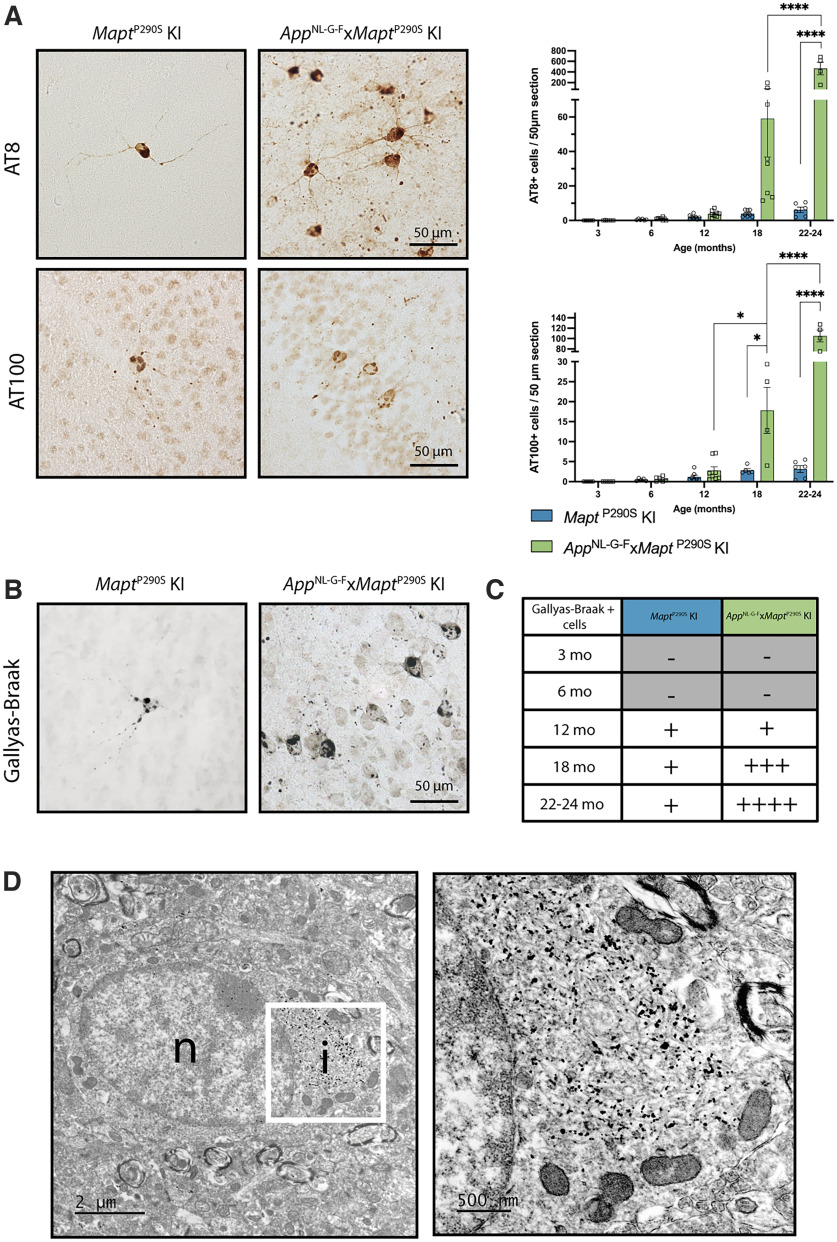
Characterization of brain tau pathology by immunohistochemistry and Gallyas-Braak silver staining. ***A***, AT8 (top) and AT100 (bottom) immunoreactivity in *Mapt*^P290S^ KI and *App*^NL-G-F^*xMapt*^P290S^ KI mice at 24 months of age. AT8- or AT100-immunoreactive cells were quantified at 3, 6, 12, 18, and 22–24 months in 50 μm whole-brain coronal sections. Results shown are the mean ± SEM. Significance between genotypes and adjacent time-points within a genotype is reported for two-way ANOVA with Tukey’s *post hoc* analysis (*p < 0.05, **p < 0.01, ****p < 0.0001). Quantitation values of AT8 staining in *Mapt*^P290S^ KI mice were as follows: 3 months, *n* = 6 (3 M, 3 F); 6 months, *n* = 6 (3 M, 3 F); 12 months, *n* = 8 (4 M, 4 F), 18 months, *n* = 8 (4 M, 4 F); and 22–24 months, *n* = 6 (3 M, 3 F). Values in *App*^NL-G-F^*xMapt*^P290S^ KI mice were as follows: 3 months, *n* = 6 (3 M, 3 F); 6 months, *n* = 8 (4 M, 4 F); 12 months, *n* = 8 (4 M, 4 F); 18 months, *n* = 8 (4 M, 4 F); 22–24 months, *n* = 4 (2 M, 2F). Brain sections from 4 (2 M, 2 F) 18-month-old *App*^NL-G-F^*xMapt*^P290S^ KI mice were not available at the time of AT100 staining. ***B***, Representative images of Gallyas-Braak silver staining at 24 months of age in *Mapt*^P290S^ and *App*^NL-G-F^*xMapt*^P290S^ KI mice. ***C***, Timeline depicting the appearance of cells labeled with Gallyas-Braak silver stain in *Mapt*^P290S^ KI and *App*^NL-G-F^*xMapt*^P290S^ KI mice. Values were as follows: at 3 and 6 months, *n* = 3 (2 M, 1 F); at 12 and 18 months, *n* = 8 (4 M, 4 F); at 22–24 months, *n* = 4 (2 M, 2 F). ***D***, *In situ* electron microscopy of Gallyas-Braak silver-stained brain sections from 24-month-old *App*^NL-G-F^*xMapt*^P290S^ KI mouse showing labeled filamentous content of a tau inclusion (i) within the cell body of a neuron, adjacent to a nucleus (n).

Antibodies AT8 and AT100 were used to assess the presence of hyperphosphorylated and assembled tau in the brains of *Mapt*^P290S^ KI and *App*^NL-G-F^ KI*xMapt*^P290S^ KI mice (Fig. 3*A*). Immunoreactivity with AT8 and AT100 became detectable at 6 months of age in both lines and increased until 12 months, mostly along midline structures. Tau inclusions were present in nerve cell bodies and axons. There were no significant differences between lines. However, between 12 and 18 months, a significant increase in the number of AT100-immunoreactive cells was present in *App*^NL-G-F^*xMapt*^P290S^ (*p* < 0.05), but not in *Mapt*^P290S^ (*p* > 0.9999), KI mice. At 18 months, there were six times as many AT100-immunoreactive cells in *App*^NL-G-F^*xMapt*^P290S^ mice than in *Mapt*^P290S^ KI mice (*p* < 0.05). There was no significant difference in the number of AT8-immunoreactive cells between *App*^NL-G-F^*xMapt*^P290S^ and *Mapt*^P290S^ KI mice, probably because of the large variability in the amount of tau pathology at this time point in the double-KI line. At 22–24 months, the difference in the number of tau inclusions between *App*^NL-G-F^*xMapt*^P290S^ and *Mapt*^P290S^ KI mice reached 33-fold with AT100 (*p* < 0.0001) and 75-fold with AT8 (*p* < 0.0001; Fig. 3*A*). Gallyas-Braak silver-positive inclusions were first detected in both KI lines at 12 months, but increased progressively until 24 months of age only in the *App*^NL-G-F^*xMapt*^P290S^ KI line (Fig. 3*B*,*C*). *In situ* transmission electron microscopy of Gallyas-Braak silver-stained tissues showed filamentous structures in nerve cells, as illustrated for a cell body from the piriform cortex (Fig. 3*D*).

### Region-specific increase in tau pathology in *App*^NL-G-F^ KI*xMapt*^P290S^ KI mice

**Figure 4.**
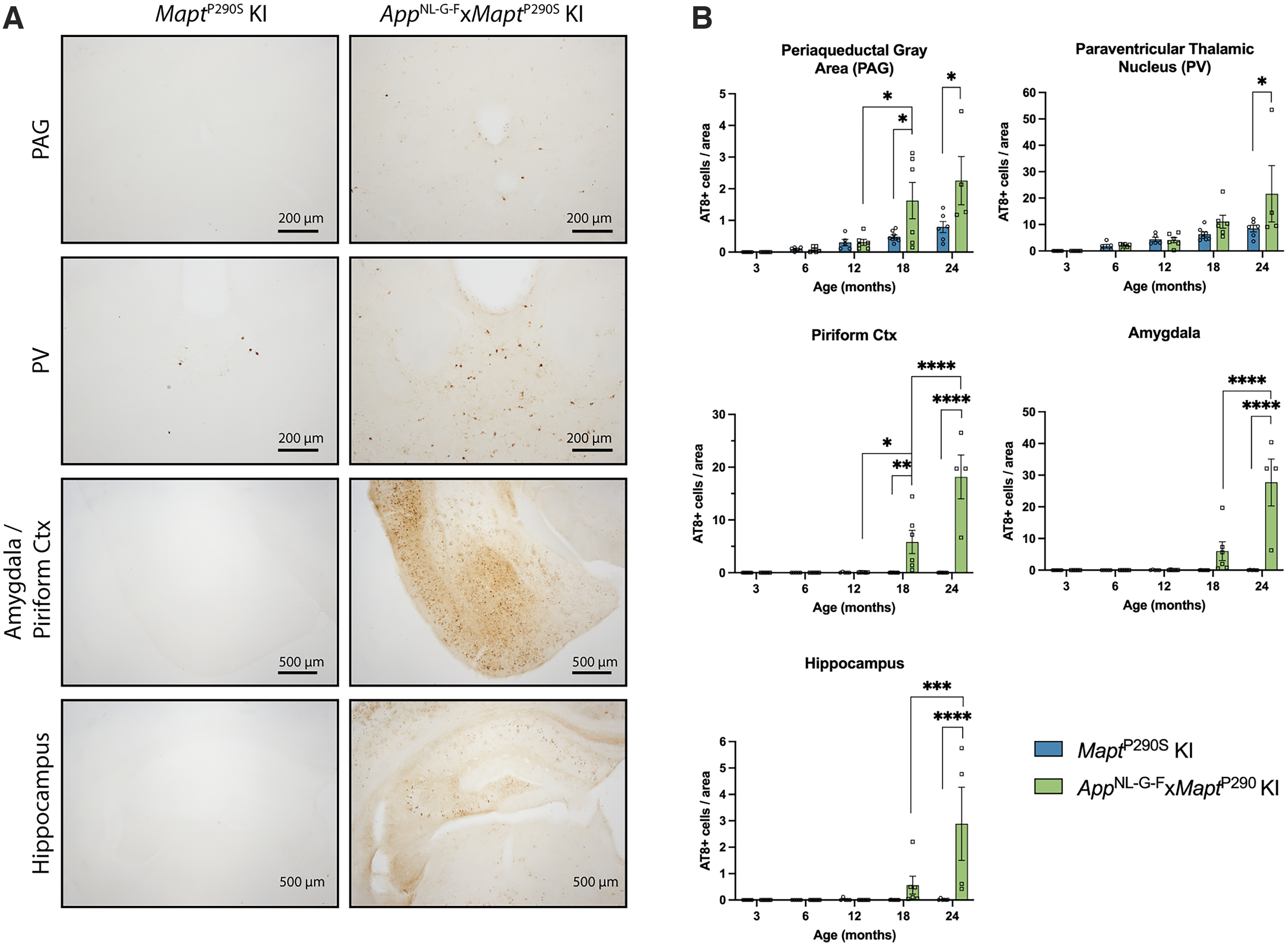
Regional quantitation of AT8-immunoreactive tau pathology. ***A***, AT8 immunoreactivity in PAG, PV, amygdala, piriform cortex, and hippocampus of 24-month-old *Mapt*^P290S^ KI (left) and *App*^NL-G-F^*xMapt*^P290S^ KI (right) mice. ***B***, Regional quantification of AT8-immunoreactive cells at 3, 6, 12, 18, and 22–24 months. Results shown are the mean ± SEM. Significance between genotypes and adjacent time-points within a genotype are reported for two-way ANOVA with Tukey’s *post hoc* analysis. *p < 0.05, **p < 0.01, ***p < 0.001, ****p < 0.0001. At 3, 6, 12, and 18 months, *n* = 6 (3 M, 3 F); at 22–24 months, *n* = 4 (2 M, 2 F).

AT8 immunoreactivity was quantified in PV, PAG, amygdala, hippocampus, and piriform cortex. In *Mapt*^P290S^ KI mice, AT8-immunoreactive cells were present in PV and PAG, but only rarely in hippocampus, amygdala, and piriform cortex. In *App*^NL-G-F^*xMapt*^P290S^ KI mice, AT8-positive cells were also present in PV and PAG; from 18 months, immunoreactivity expanded into amygdala, hippocampus, and piriform cortex (Fig. 4*A*). At 18 months of age, there was a threefold increase in AT8-immunoreactive cells in the PAG (*p* = 0.0405), with no significant difference in the PV, and a 33-fold increase in the piriform cortex (*p* = 0.0086) of *App*^NL-G-F^*xMapt*^P290S^ KI mice, compared with age-matched *Mapt*^P290S^ KI mice. At 24 months, this increase was threefold in PAG (*p* = 0.0206) and PV (*p* = 0.0377), and at least 170-fold in the amygdala (*p* < 0.0001), hippocampus (*p* = 0.0005), and piriform cortex (*p* < 0.0001; Fig. 4*B*). Cerebellum, striatum, and lumbar spinal cord were devoid of tau immunoreactivity.

### Neuritic plaques with and without tau filaments

**Figure 5.**
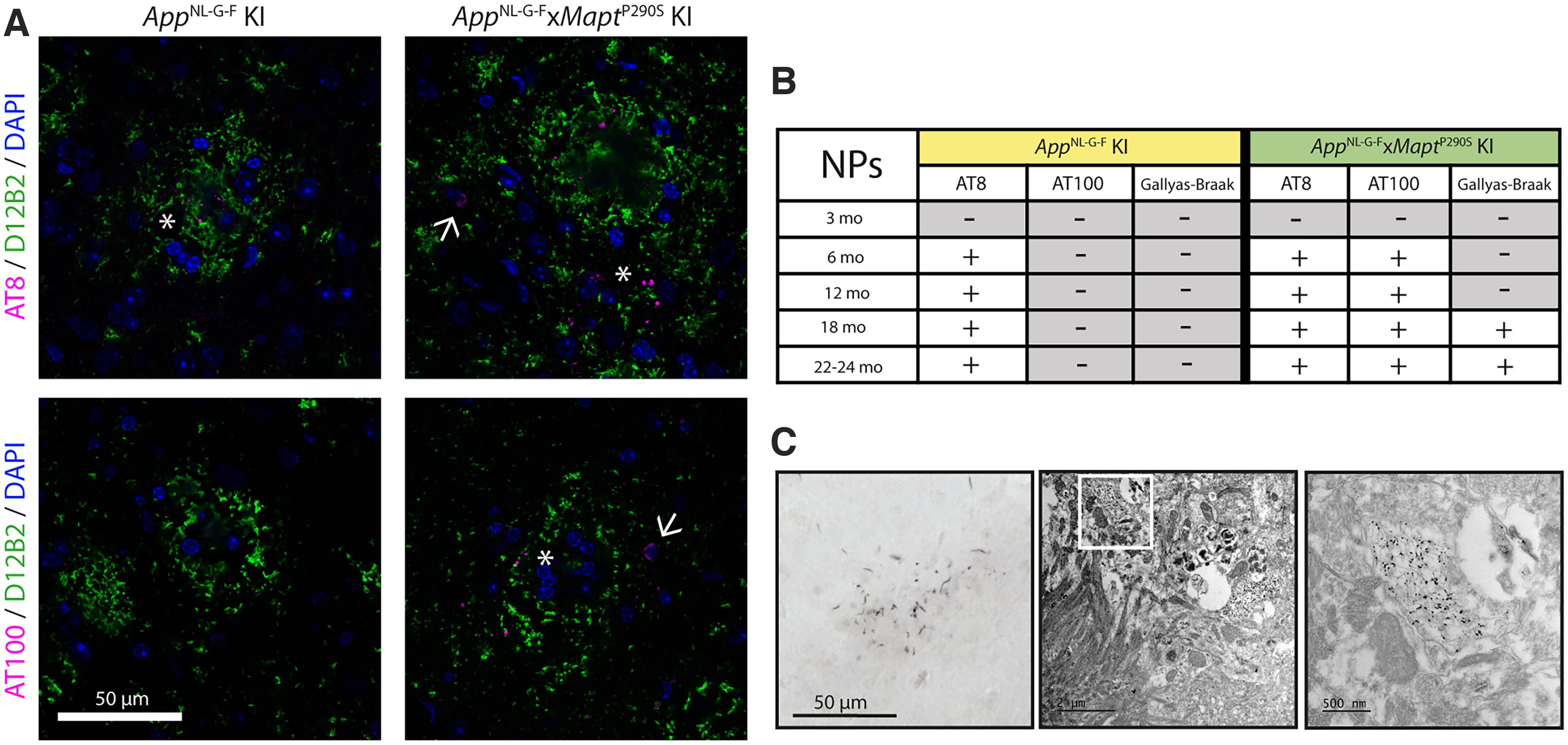
Neuritic plaques in *Mapt*^P290S^ and *App*^NL-G-F^*xMapt*^P290S^ KI mice. ***A***, Immunofluorescence staining of tau (AT8 and AT100) and Aβ (D12B2) in 18-month-old *App*^NL-G-F^ and *App*^NL-G-F^*xMapt*^P290S^ KI mouse brains. *App*^NL-G-F^ brains contain neuritic plaques (asterisk) that are positive for AT8, but not for AT100. *App*^NL-G-F^*xMapt*^P290S^ KI KI brains contain neuritic plaques and tau inclusions (arrow) that are positive for both AT8 and AT100. ***B***, Timeline of the appearance of neuritic plaque labeled with AT8, AT100, and Gallyas-Braak silver staining in *App*^NL-G-F^and *App*^NL-G-F^*xMapt*^P290S^ KI lines. Values are as follows: at 3 and 6 months, *n* = 3 (2 M, 1 F); at 12 and 18 months, *n* = 8 (4 M, 4 F); at 22–24 months, *n* = 4 (2 M, 2 F). ***C***, Gallyas-Braak-labeled neuritic plaque in the piriform cortex of an *App*^NL-G-F^*xMapt*^P290S^ KI mouse at 24 months of age, visualized by light microscopy and *in* situ electron microscopy.

In the brains from *App*^NL-G-F^ KI mice, Aβ plaques were surrounded by dystrophic neurites from 6 months of age onward; they were immunoreactive with AT8, but negative with AT100 and Gallyas-Braak silver. In contrast, in *App*^NL-G-F^*xMapt*^P290S^ KI mice, neuritic plaques were immunoreactive with AT8 and AT100 from 6 months of age onward (Fig. 5*A*,*B*). They were Gallyas-Braak silver positive at 18 months (Fig. 5*B*,*C*). This shows that dystrophic neurites from *App*^NL-G-F^*xMapt*^P290S^ mice contained hyperphosphorylated and filamentous tau, whereas in neuritic plaques from *App*^NL-G-F^ *KI* mice, tau was hyperphosphorylated, but not filamentous. By *in situ* transmission electron microscopy of Gallyas-Braak silver-stained brain tissues from *App*^NL-G-F^*xMapt*^P290S^ mice, neuritic processes with labeled filaments surrounded Aβ plaques (Fig. 5*C*).

### Seeded assembly of tau

**Figure 6.**
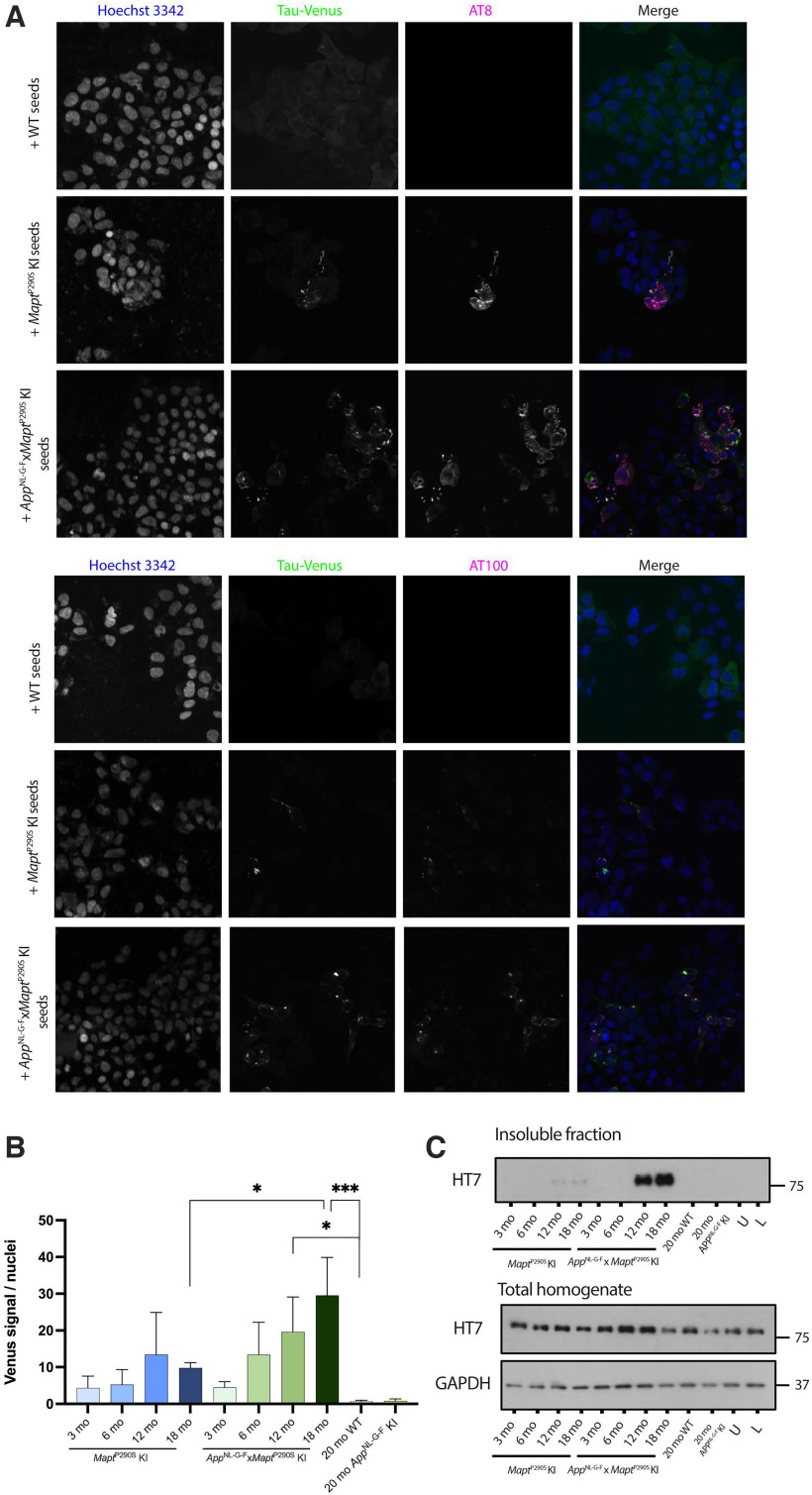
Tau assemblies extracted from brains of *Mapt*^P290S^ and *App*^NL-G-F^*xMapt*^P290S^ KI mice induce aggregation of P301S tau expressed in HEK293 cells. ***A***, Representative images of HEK293 P301S tau-venus cells seeded with 18-month-old *Mapt*^P290S^ and *App*^NL-G-F^*xMapt*^P290S^ Sarkosyl-insoluble tau assemblies labeled with AT8 and AT100. ***B***, Quantitation of tau-venus signal per nucleus seeded with assemblies from 3-, 6-, 12-, and 18-month-old *Mapt*^P290S^ and *App*^NL-G-F^*xMapt*^P290S^ and 20-month-old wild-type and *App*^NL-G-F^ controls (*n* = 3). ***C***, Representative immunoblots of total homogenates and Sarkosyl-insoluble extracts from seeded cells using human-tau antibody HT7 (*n* = 3).

We used a tau reporter line in HEK293 cells expressing human 0N4R P301S tau with a venus tag (McEwan et al., 2017) to investigate seeding activity, as assessed by fluorescent cytoplasmic foci, of Sarkosyl-insoluble brain extracts from *Mapt*^P290S^ and *App*^NL-G-F^*xMapt*^P290S^ KI mice. Sarkosyl-insoluble brain extracts from 18-month-old *Mapt*^P290S^ and *App*^NL-G-F^*xMapt*^P290S^ KI mice seeded, as evidenced by the presence of AT8- and AT100-positive fluorescent puncta (Fig. 6*A*). Seeding with brain extracts from *Mapt*^P290S^ KI mice was higher than with brain extracts from wild-type mice, but this difference was not statistically significant. The seeding ability of brain extracts from *App*^NL-G-F^*xMapt*^P290S^ mice was 28-fold higher than that of wild-type brain extracts at 12 months (*p* = 0.0440), and 43-fold higher than that of wild-type brain extracts at 18 months of age (*p* < 0.0001). At 18 months, there was a significant threefold difference in seeding activity between *App*^NL-G-F^*xMapt*^P290S^ and *Mapt*^P290S^ KI mice (Fig. 6*B*). Immunoblotting with an anti-human tau antibody showed that small amounts of Sarkosyl-insoluble tau could be extracted from cells seeded with brain extracts from 12- and 18-month-old *Mapt*^P290S^ KI mice, indicating that low levels of seed-competent tau were present, in accordance with these mice having little tau pathology. Brain extracts from *App*^NL-G-F^*xMapt*^P290S^ KI mice, on the other hand, seeded more insoluble tau, consistent with the presence of increased tau pathology (Fig. 6*C*).

### Age-dependent neurodegeneration in *App*^NL-G-F^*xMapt*^P290S^ KI mice

**Figure 7.**
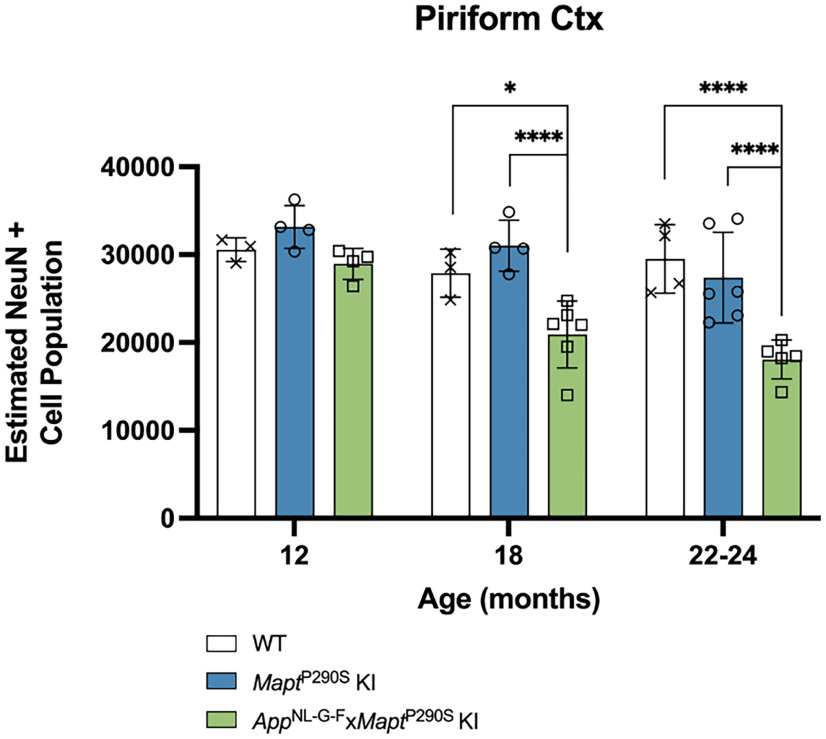
Age-dependent neurodegeneration in *App*^NL-G-F^*xMapt*^P290S^ KI mice. Quantitation of NeuN-positive cells in piriform cortex of wild-type, *Mapt*^P290S^ KI, and *App*^NL-G-F^*xMapt*^P290S^ KI mice at 12, 18, and 22–24 months of age. Results shown are the mean ± SEM. *p < 0.05, ****p < 0.0001 for two-way ANOVA with Tukey’s *post hoc* analysis. For wild-type mice at all ages, *n* = 3 (M); for *Mapt*^P290S^ KI mice at 12 and 18 months, *n* = 4 (2 M, 2 F); at 22–24 months, *n* = 6 (3 M, 3 F); for *App*^NL-G-F^*xMapt*^P290S^ KI at 12 and 18 months, *n* = 4 (2 M, 2 F); at 22–24 months *n* = 5 (2 M, 3 F).

Nerve cell loss in the piriform cortex was quantified using unbiased stereological cell counting. Significant nerve cell loss in *App*^NL-G-F^*xMapt*^P290S^ compared with age-matched wild-type (*p* = 0.0178) and *Mapt*^P290S^ KI mice (*p* = 0.0002) was detected from 18 months of age onward (Fig. 7*A*). No significant nerve cell loss was detected in the *Mapt*^P290S^ KI mice compared with age-matched wild-type mice.

## Discussion

We report the production and characterization of a knock-in mouse model (*Mapt*^P290S^ KI), expressing mutant murine tau at endogenous levels. In contrast to the P290L knock-in line, in which tau pathology could not be detected by AT8 or Gallyas-Braak staining until 32 months of age, the longest time point studied (Gilley et al., 2012), *Mapt^P^*^290S^ KI mice exhibited a limited number of AT100-positive tau inclusions within a restricted group of midline nuclei from 6 months of age. However, Sarkosyl-insoluble tau was not detected by immunoblotting, probably because of the small number of inclusions. In view of AT100 positivity (Delobel et al., 2008), these findings suggest that murine tau can assemble *in vivo*, despite differences in isoform composition and amino acid sequence with human tau. Mice express 3R tau during fetal development, but only 4R tau is found in the brains of adults (Brion et al., 1993; Gotz et al., 1995). Additional differences between murine and human tau have been described in the N terminus (Hernandez et al., 2020). Maintaining the murine *Mapt* gene may be a desirable alternative to the humanization of tau expressed in a murine environment, since murine binding partners may be unable to interact with human tau isoforms. Mouse lines expressing all six isoforms of humanized wild-type tau at endogenous levels have been produced (Saito et al., 2019; He et al., 2020). These mice did not develop overt tau pathology, and crossing them with *App*^NL-G-F^ KI mice led to increased tau phosphorylation, but tau inclusions were not detected for up to 24 months (Saito et al., 2019). A lack of tau pathology suggests that humanization of wild-type tau is insufficient to induce tau pathology, even in the presence of Aβ. Pathogenic mutations may be required to study aggregation-prone tau in mice.

By crossing our *Mapt*^P290S^ KI mice with the *App*^NL-G-F^ KI line (*App*^NL-G-F^*xMapt*^P290S^ KI), we demonstrate that Aβ pathology promoted tau aggregation in an age-dependent manner. Sarkosyl-insoluble tau was only detectable by immunoblotting in 18-month-old *App*^NL-G-F^*xMapt*^P290S^ KI mice, and significantly more tau inclusions were found in 18-month-old *App*^NL-G-F^*xMapt*^P290S^ KI mice compared with age-matched *Mapt*^P290S^ KI mice. This is in agreement with overexpression models, which showed enhanced tau pathology in the presence of Aβ (Lewis et al., 2001; Chen et al., 2016). Unlike these models, which exhibited significant tau pathology at a young age and often needed to be culled before 12 months, the small number of tau inclusions in *Mapt*^P290S^ KI mice enabled us to model interactions between tau inclusions and Aβ plaques as a function of age. Mice older than 18 months are considered “elderly” (Flurkey, 2007) and begin to exhibit signs of cognitive impairment at ∼22 months (Yanai and Endo, 2021). Consistent with an effect of aging, we only observed a significant effect of Aβ on the number of tau inclusions after 18 months, despite Aβ deposition beginning as early as 3 months.

In AD, tau pathology propagates through the brain in a stereotypical manner. Tau inclusions develop in the entorhinal cortex and later progress to limbic areas, followed by the neocortex (Braak and Braak, 1991). Overexpression models rely on the expression of tau under the control of strong promoters, such as murine *Thy-1* (Allen et al., 2002) and prion protein promoters (Lewis et al., 2000; 2001). This leads to tau pathology developing in spinal cord, with early behavioral phenotypes affecting motor function and gait. With mutant tau under the control of its endogenous promoter, we instead observed only minimal tau pathology along midline structures in *Mapt*^P290S^ KI brains, in the absence of motor dysfunction. Interestingly, in the presence of Aβ, tau pathology appeared in regions reminiscent of Braak staging, such as entorhinal cortex, hippocampus, and amygdala, as well as piriform cortex. This is consistent with a recent longitudinal amyloid and tau PET study, which found that people with Aβ plaques in the entorhinal cortex are more likely to have spreading of tau pathology on follow-up (Lee, Brown, et al., 2022).

Dystrophic neurites that are AT8-positive have been reported to surround Aβ plaques in *App*^NL-G-F^ KI mice, with increased AT8 signal in double-KI mice expressing all six humanized tau isoforms (Saito et al., 2019). However, assembled tau was not present in those dystrophic neurites. We report dystrophic neurites in *App*^NL-G-F^*xMapt*^P290S^ KI mouse brains that were AT8, AT100, and Gallyas-Braak positive, indicating the presence of filamentous tau. In support, we observed silver-positive filaments in neurites surrounding Aβ plaques in brain sections from 24-month-old *App*^NL-G-F^*xMapt*^P290S^ KI mice by *in situ* electron microscopy. Surprisingly, we observed that amyloid plaques appeared to form intracellularly within an enclosing membrane. However, in instances where these membranes ruptured, plaques were surrounded by Gallyas-Braak-positive neuritic processes, astrocytic processes, and many microglial cells. These observations are consistent with the recent description of PANTHOS in APP transgenic mice and AD brains (Lee et al., 2022).

The role of neuritic plaques in the propagation of tau pathology remains unclear. Intracerebral injection of extracts from AD brains into 5xFAD mice with significant Aβ pathology led to the formation of dystrophic neurites and seeded endogenous tau inclusions (Vergara et al., 2019). Studies of AD extract-injected *App*^NL-G-F^ KI mice have also suggested that the environment of Aβ plaques facilitates seeded assembly and propagation of endogenous tau (He et al., 2018).

We used a cell-based model to investigate the seeding ability of tau filaments extracted from *Mapt*^P290S^ KI and *App*^NL-G-F^*xMapt*^P290S^ KI brains (McEwan et al., 2017). We were able to detect seeded tau from cells treated with both aged *Mapt*^P290S^ KI and *App*^NL-G-F^*xMapt*^P290S^ KI brain extracts. This suggests that seed-competent tau is present in aged *Mapt*^P290S^ KI brains, but significant propagation of tau pathology did not occur *in vivo* in the absence of Aβ plaques. There is growing evidence that Aβ pathology promotes the spreading of tau pathology. Tau propagation to the entorhinal cortex of mice overexpressing human P301L tau was accelerated when crossed with APP/PS1 mice (Pooler et al., 2015). Brain homogenates from tauopathy patients showed enhanced seeding ability in cases with both tau neurofibrillary tangles and Aβ plaques, compared with cases without plaques (Bennett et al., 2017). Immunotherapy aimed at reducing Aβ plaque burden also appears to have an effect on tau spreading. In 3xTg-AD mice, anti-Aβ immunotherapy has been shown to reduce the amount of tau pathology (Oddo et al., 2004). A recent report of an 84-year-old woman receiving the anti-Aβ antibody aducanumab for 32 months showed lower phospho-tau immunoreactivity when compared with untreated AD cases (Plowey et al., 2022). Together, these findings suggest that assembled Aβ can promote, but not induce, the assembly of tau. The same conclusion was reached in studies of tau filaments from human brains by electron cryomicroscopy (Shi et al., 2021).

To look for neurodegeneration, we examined the piriform cortex of KI mice using unbiased stereological cell counting. We observed nerve cell loss in 18- to 24-month-old *App*^NL-G-F^*xMapt*^P290S^ KI mouse brains, coinciding with the significant amplification of tau inclusions and the presence of tau filaments. The relationship between filamentous tau assemblies and nerve cell loss has been characterized in an overexpressing model of P301S tau pathology (Macdonald et al., 2019). Our findings extend the correlation between filamentous tau pathology and neurodegeneration to a mouse model expressing endogenous levels of tau.

We propose that *App*^NL-G-F^*xMapt*^P290S^ KI mice provide a good model system for studying the interactions among aggregation-prone tau, Aβ, neuritic plaques, neurodegeneration, and aging. Despite this, there are limitations to this model as a representation of sporadic AD, which is characterized by the aggregation of wild-type tau and Aβ. Humans with the P301S mutation in tau, together with a mutation in APP, have not been reported. These mutations probably affect the conformation of protein aggregates, and the current model should be used bearing this in mind. Current evidence suggests, however, that mice do not develop wild-type tau aggregates without significant genetic manipulation. In the presence of Aβ plaques, humanized wild-type tau does not aggregate into mature neurofibrillary tangles in aged mouse brain (Saito et al., 2019). It is thus likely that a certain degree of genetic manipulation of *Mapt* and *App* is required for modeling tau and Aβ pathologies in mice. In this case, the *App*^NL-G-F^*xMapt*^P290S^ KI mice offer advantages over models that rely on transgenic overexpression of mutant tau or ΑPP. With tau and APP expressed under the control of the endogenous murine promoter, artificial phenotypes, owing to overexpression and random insertion of transgenes, are avoided (Saito et al., 2016; Gamache et al., 2019). We suggest that the *Mapt*^P290S^*xApp*^NL-G-F^ *KI* mice are a valuable alternative model for studying tau pathologies in the context of Aβ. (The mice can be obtained by contacting our research office at techtran@mrc-lmb.cam.ac.uk.)

## Synthesis

Reviewing Editor: Karl Herrup, University of Pittsburgh School of Medicine Decisions are customarily a result of the Reviewing Editor and the peer reviewers coming together and discussing their recommendations until a consensus is reached. When revisions are invited, a fact-based synthesis statement explaining their decision and outlining what is needed to prepare a revision will be listed below. The following reviewer(s) agreed to reveal their identity: Zoltan Simandi, Kurt Brunden. Note: If this manuscript was transferred from JNeurosci and a decision was made to accept the manuscript without peer review, a brief statement to this effect will instead be what is listed below.

The work is significantly improved. The additional methods are most welcome. It is ready for publication, but one of the reviewers picked up a few very minor issues that should be fixed before going to press. No re-review will be necessary; I will approve the changes at the Editorial level. Thank you for a fine submission and a very responsive revision.

Minor reviewer concerns:

P. 7 (Methods) - The authors have provided an improved description of the backcrossing and percent background purity of the MaptP290S line, and it would be helpful if they also included in the Methods section the information provided by the Saito lab on the backcrossing of the AppNL-G-F line, as discussed in their response to reviewers.

P. 10 (Methods) and P. 35 (Figure Legend) - In describing the quantitation of brain staining, the authors state that “Mouse brain sections from 4 (2M, 2F) 18-month old mice were not available for AT100 staining”. Does this apply to both the Mapt and APP x Mapt mice, or just one of the mouse groups? This should be specified.

P. 11 (Methods) and P. 35 (Figure Legend) - The number of mice utilized for Gallyas staining is specified, but the number of each sex should also be stated.

P. 27 - The first line of the page has a typo, as “together a mutation” should be “together with a mutation”.

P. 37 (Figure Legend) - The number of each sex used for the neuritic plaque quantification should be specified.

## Author Response

Final Revisions

1. P. 7 (Methods) - The authors have provided an improved description of the backcrossing and percent background purity of the MaptP290S line, and it would be helpful if they also included in the Methods section the information provided by the Saito lab on the backcrossing of the AppNL-G-F line, as discussed in their response to reviewers.

These details have been included in the methods section on page 7.

2. P. 10 (Methods) and P. 35 (Figure Legend) - In describing the quantitation of brain staining, the authors state that “Mouse brain sections from 4 (2M, 2F) 18-month old mice were not available for AT100 staining”. Does this apply to both the Mapt and APP x Mapt mice, or just one of the mouse groups? This should be specified.

This applied to only the APPxMapt mice. We have clarified this in the methods section on page 10 and the figure legend on page 35.

3. P. 11 (Methods) and P. 35 (Figure Legend) - The number of mice utilized for Gallyas staining is specified, but the number of each sex should also be stated.

This has been amended on pages 11 and 35.

4. P. 27 - The first line of the page has a typo, as “together a mutation” should be “together with a mutation”.

This has been amended on page 27.

5. P. 37 (Figure Legend) - The number of each sex used for the neuritic plaque quantification should be specified.

This has been amended on page 37.

## References

[B1] Allen B, Ingram E, Takao M, Smith MJ, Jakes R, Virdee K, Yoshida H, Holzer M, Craxton M, Emson PC, Atzori C, Migheli A, Crowther RA, Ghetti B, Spillantini MG, Goedert M (2002) Abundant tau filaments and nonapoptotic neurodegeneration in transgenic mice expressing human P301S tau protein. J Neurosci 22:9340–9351. 10.1523/JNEUROSCI.22-21-09340.2002 12417659 PMC6758022

[B2] Bennett RE, DeVos SL, Dujardin S, Corjuc B, Gor R, Gonzalez J, Roe AD, Frosch MP, Pitstick R, Carlson GA, Hyman BT (2017) Enhanced tau aggregation in the presence of amyloid beta. Am J Pathol 187:1601–1612. 28500862 10.1016/j.ajpath.2017.03.011 PMC5500829

[B39] Braak H, Del Tredici K (2015) Neuroanatomy and pathology of sporadic Alzheimer’s disease. Adv Anat Embryol Cell Biol 215:1–162. 25920101

[B3] Braak H, Braak E (1991) Neuropathological stageing of Alzheimer-related changes. Acta Neuropathol 82:239–259. 1759558 10.1007/BF00308809

[B4] Brion JP, Smith C, Couck AM, Gallo JM, Anderton BH (1993) Developmental changes in tau phosphorylation: fetal tau is transiently phosphorylated in a manner similar to paired helical filament-tau characteristic of Alzheimer's disease. J Neurochem 61:2071–2080. 10.1111/j.1471-4159.1993.tb07444.x 8245963

[B5] Bugiani O, Murrell JR, Giaccone G, Hasegawa M, Ghigo G, Tabaton M, Morbin M, Primavera A, Carella F, Solaro C, Grisoli M, Savoiardo M, Spillantini MG, Tagliavini F, Goedert M, Ghetti B (1999) Frontotemporal dementia and corticobasal degeneration in a family with a P301S mutation in tau. J Neuropathol Exp Neurol 58:667–677. 10374757 10.1097/00005072-199906000-00011

[B6] Chen W, Abud EA, Yeung ST, Lakatos A, Nassi T, Wang J, Blum D, Buee L, Poon WW, Blurton-Jones M (2016) Increased tauopathy drives microglia-mediated clearance of beta-amyloid. Acta neuropathol commun 4:63. 10.1186/s40478-016-0336-1 27339073 PMC4918195

[B38] Delobel P, Lavenir I, Fraser G, Ingram E, Holzer M, Ghetti B, Spillantini MG, Crowther RA, Goedert M (2008) Analysis of tau phosphorylation and truncation in a mouse model of human tauopathy. Am J Pathol 172:123–131. 18079436 10.2353/ajpath.2008.070627 PMC2189621

[B7] Flurkey K, Currer JM, Harrison DE (2007) Mouse models in aging research. In: The mouse in biomedical research: history, wild mice, and genetics, Ed 2 (Barthold SW, Fox JG, Davisson MT, Newcomer CE, Quimby FW, Smith AL, eds), pp 637–672. Burlington, MA: Academic.

[B8] Franklin KBJ, Paxinos G (2013) Paxinos and Franklin's the mouse brain in stereotaxic coordinates. Amsterdam: Academic.

[B9] Gamache J, Benzow K, Forster C, Kemper L, Hlynialuk C, Furrow E, Ashe KH, Koob MD (2019) Factors other than hTau overexpression that contribute to tauopathy-like phenotype in rTg4510 mice. Nat Commun 10:2479. 10.1038/s41467-019-10428-1 31171783 PMC6554306

[B10] Gilley J, Seereeram A, Ando K, Mosely S, Andrews S, Kerschensteiner M, Misgeld T, Brion JP, Anderton B, Hanger DP, Coleman MP (2012) Age-dependent axonal transport and locomotor changes and tau hypophosphorylation in a “P301L” tau knockin mouse. Neurobiology of Aging 33:621.e1–621.e15. 10.1016/j.neurobiolaging.2011.02.014 21492964

[B11] Goedert M, Spillantini MG, Cairns NJ, Crowther RA (1992) Tau-proteins of Alzheimer paired helical filaments: abnormal phosphorylation of all 6 brain isoforms. Neuron 8:159–168. 10.1016/0896-6273(92)90117-V 1530909

[B12] Gotz J, Probst A, Spillantini MG, Schafer T, Jakes R, Burki K, Goedert M (1995) Somatodendritic localization and hyperphosphorylation of tau protein in transgenic mice expressing the longest human brain tau isoform. EMBO J 14:1304–1313. 7729409 10.1002/j.1460-2075.1995.tb07116.x PMC398215

[B36] Hardy JA, Higgins GA (1992) Alzheimers-Disease - the Amyloid Cascade Hypothesis. Science 256:184–185. 1566067 10.1126/science.1566067

[B37] Hardy J, Selkoe DJ (2002) The amyloid hypothesis of Alzheimer’s disease: progress and problems on the road to therapeutics. Science 297:353–356. 12130773 10.1126/science.1072994

[B13] He Z, Guo JL, McBride JD, Narasimhan S, Kim H, Changolkar L, Zhang B, Gathagan RJ, Yue C, Dengler C, Stieber A, Nitla M, Coulter DA, Abel T, Brunden KR, Trojanowski JQ, Lee VM (2018) Amyloid-beta plaques enhance Alzheimer's brain tau-seeded pathologies by facilitating neuritic plaque tau aggregation. Nat Med 24:29–38. 29200205 10.1038/nm.4443 PMC5760353

[B14] He Z, McBride JD, Xu H, Changolkar L, Kim SJ, Zhang B, Narasimhan S, Gibbons GS, Guo JL, Kozak M, Schellenberg GD, Trojanowski JQ, Lee VM (2020) Transmission of tauopathy strains is independent of their isoform composition. Nat Commun 11:7. 31911587 10.1038/s41467-019-13787-x PMC6946697

[B15] Hernandez F, Merchan-Rubira J, Valles-Saiz L, Rodriguez-Matellan A, Avila J (2020) Differences between human and murine tau at the N-terminal end. Front Aging Neurosci 12:11. 32063841 10.3389/fnagi.2020.00011 PMC6999090

[B16] Ishihara T, Hong M, Zhang B, Nakagawa Y, Lee MK, Trojanowski JQ, Lee VM (1999) Age-dependent emergence and progression of a tauopathy in transgenic mice overexpressing the shortest human tau isoform. Neuron 24:751–762. 10595524 10.1016/s0896-6273(00)81127-7

[B17] Lee JH, Yang DS, Goulbourne CN, Im E, Stavrides P, Pensalfini A, Chan H, Bouchet-Marquis C, Bleiwas C, Berg MJ, Huo C, Peddy J, Pawlik M, Levy E, Rao M, Staufenbiel M, Nixon RA (2022) Faulty autolysosome acidification in Alzheimer's disease mouse models induces autophagic build-up of Abeta in neurons, yielding senile plaques. Nat Neurosci 25:688–701. 35654956 10.1038/s41593-022-01084-8 PMC9174056

[B18] Lee WJ, Brown JA, Kim HR, La Joie R, Cho H, Lyoo CH, Rabinovici GD, Seong JK, Seeley WW (2022) Regional Aβ-tau interactions promote onset and acceleration of Alzheimer's disease tau spreading. Neuron 110:1932–1943.e5. 10.1016/j.neuron.2022.03.034 35443153 PMC9233123

[B19] Lewis J, McGowan E, Rockwood J, Melrose H, Nacharaju P, Van Slegtenhorst M, Gwinn-Hardy K, Paul Murphy M, Baker M, Yu X, Duff K, Hardy J, Corral A, Lin WL, Yen SH, Dickson DW, Davies P, Hutton M (2000) Neurofibrillary tangles, amyotrophy and progressive motor disturbance in mice expressing mutant (P301L) tau protein. Nat Genet 25:402–405. 10932182 10.1038/78078

[B20] Lewis J, Dickson DW, Lin WL, Chisholm L, Corral A, Jones G, Yen SH, Sahara N, Skipper L, Yager D, Eckman C, Hardy J, Hutton M, McGowan E (2001) Enhanced neurofibrillary degeneration in transgenic mice expressing mutant tau and APP. Science 293:1487–1491. 11520987 10.1126/science.1058189

[B21] Liu H, Wang W, Chew SK, Lee SC, Li J, Vassiliou GS, Green T, Futreal PA, Bradley A, Zhang S, Liu P (2011) Stella-Cre mice are highly efficient Cre deleters. Genesis 49:689–695. 21786403 10.1002/dvg.20741 PMC3674523

[B22] Macdonald JA, Bronner IF, Drynan L, Fan J, Curry A, Fraser G, Lavenir I, Goedert M (2019) Assembly of transgenic human P301S Tau is necessary for neurodegeneration in murine spinal cord. Acta Neuropathol Commun 7:44. 10.1186/s40478-019-0695-5 30885267 PMC6421678

[B23] McEwan WA, Falcon B, Vaysburd M, Clift D, Oblak AL, Ghetti B, Goedert M, James LC (2017) Cytosolic Fc receptor TRIM21 inhibits seeded tau aggregation. Proc Natl Acad Sci U S A 114:574–579. 10.1073/pnas.1607215114 28049840 PMC5255578

[B24] Oddo S, Billings L, Kesslak JP, Cribbs DH, LaFerla FM (2004) A beta immunotherapy leads to clearance of early, but not late, hyperphosphorylated tau aggregates via the proteasome. Neuron 43:321–332. 10.1016/j.neuron.2004.07.003 15294141

[B25] Plowey ED, Bussiere T, Rajagovindan R, Sebalusky J, Hamann S, von Hehn C, Castrillo-Viguera C, Sandrock A, Budd Haeberlein S, van Dyck CH, Huttner A (2022) Alzheimer disease neuropathology in a patient previously treated with aducanumab. Acta Neuropathol 144:143–153. 10.1007/s00401-022-02433-4 35581440 PMC9217863

[B26] Pooler AM, Polydoro M, Maury EA, Nicholls SB, Reddy SM, Wegmann S, William C, Saqran L, Cagsal-Getkin O, Pitstick R, Beier DR, Carlson GA, Spires-Jones TL, Hyman BT (2015) Amyloid accelerates tau propagation and toxicity in a model of early Alzheimer's disease. Acta Neuropathol Commun 3:14. 10.1186/s40478-015-0199-x 25853174 PMC4371800

[B27] Radde R, Bolmont T, Kaeser SA, Coomaraswamy J, Lindau D, Stoltze L, Calhoun ME, Jaggi F, Wolburg H, Gengler S, Haass C, Ghetti B, Czech C, Holscher C, Mathews PM, Jucker M (2006) A beta 42-driven cerebral amyloidosis in transgenic mice reveals early and robust pathology. Embo Rep 7:940–946. 10.1038/sj.embor.7400784 16906128 PMC1559665

[B28] Sahara N, Lewis J, DeTure M, McGowan E, Dickson DW, Hutton M, Yen SH (2002) Assembly of tau in transgenic animals expressing P301L tau: alteration of phosphorylation and solubility. J Neurochem 83:1498–1508. 12472903 10.1046/j.1471-4159.2002.01241.x

[B29] Saito T, Matsuba Y, Mihira N, Takano J, Nilsson P, Itohara S, Iwata N, Saido TC (2014) Single App knock-in mouse models of Alzheimer's disease. Nat Neurosci 17:661–663. 10.1038/nn.3697 24728269

[B30] Saito T, Matsuba Y, Yamazaki N, Hashimoto S, Saido TC (2016) Calpain activation in Alzheimer's model mice is an artifact of APP and presenilin overexpression. J Neurosci 36:9933–9936. 27656030 10.1523/JNEUROSCI.1907-16.2016 PMC5030353

[B31] Saito T, Mihira N, Matsuba Y, Sasaguri H, Hashimoto S, Narasimhan S, Zhang B, Murayama S, Higuchi M, Lee VMY, Trojanowski JQ, Saido TC (2019) Humanization of the entire murine Mapt gene provides a murine model of pathological human tau propagation. J Biol Chem 294:12754–12765. 31273083 10.1074/jbc.RA119.009487 PMC6709628

[B35] Shi Y et al. (2021) Structure-based classification of tauopathies. Nature 598:359–363. 34588692 10.1038/s41586-021-03911-7 PMC7611841

[B32] Vergara C, Houben S, Suain V, Yilmaz Z, De Decker R, Vanden Dries V, Boom A, Mansour S, Leroy K, Ando K, Brion JP (2019) Amyloid-beta pathology enhances pathological fibrillary tau seeding induced by Alzheimer PHF in vivo. Acta Neuropathol 137:397–412. 30599077 10.1007/s00401-018-1953-5

[B33] Yanai S, Endo S (2021) Functional aging in male C57BL/6J mice across the life-span: a systematic behavioral analysis of motor, emotional, and memory function to define an aging phenotype. Front Aging Neurosci 13:697621. 34408644 10.3389/fnagi.2021.697621 PMC8365336

[B34] Yoshiyama Y, Higuchi M, Zhang B, Huang S-M, Iwata N, Saido TC, Maeda J, Suhara T, Trojanowski JQ, Lee VM-Y (2007) Synapse loss and microglial activation precede tangles in a P301S tauopathy mouse model. Neuron 53:337–351. 10.1016/j.neuron.2007.01.010 17270732

